# Retrospective Evaluation of the Implementation of Universal Suicide Risk Screening for Youth in the Perioperative and Procedural Areas of a Health System

**DOI:** 10.1002/pan.70127

**Published:** 2026-01-14

**Authors:** Michelle Tsao, Arkadeep Ghosh, Amanda N. Burnside, Chunyi Wu, Matthew J. Rowland, Eric Cheon, Jennifer A. Hoffmann

**Affiliations:** ^1^ Department of Pediatric Anesthesiology Ann & Robert H. Lurie Children's Hospital of Chicago Chicago Illinois USA; ^2^ Northwestern University Feinberg School of Medicine Chicago Illinois USA; ^3^ University of Missouri‐Kansas City, School of Medicine Kansas City Missouri USA; ^4^ The Pritzker Department of Psychiatry and Behavioral Health Ann & Robert H. Lurie Children's Hospital of Chicago Chicago Illinois USA; ^5^ Division of Critical Care Medicine Ann & Robert H. Lurie Children's Hospital of Chicago Chicago Illinois USA; ^6^ Division of Emergency Medicine Ann & Robert H. Lurie Children's Hospital of Chicago Chicago Illinois USA; ^7^ Department of Pediatrics and Medical Social Sciences Northwestern University Feinberg School of Medicine Chicago Illinois USA

**Keywords:** adolescent, perioperative care, suicide

## Abstract

**Background:**

Suicide is a leading cause of death among US youth. Universal suicide risk screening can identify youth with unmet mental health needs, enabling interventions and linkage to services, yet screening is not widely implemented in perioperative and procedural settings.

**Aims:**

We retrospectively assessed implementation of universal suicide risk screening for youth in perioperative and procedural areas of a pediatric health system by examining screening completion rates, positivity rates, and demographic and clinical factors associated with screening.

**Methods:**

We conducted a retrospective cross‐sectional study of universal suicide risk screening among youth 10–21 years of age in perioperative and procedural areas of an academic children's hospital and two affiliated ambulatory surgical centers, using electronic health record data from August 2022 to February 2025. We described rates of screening completion using Ask Suicide‐Screening Questions (ASQ) and screening positivity rates. We used multivariable logistic regression to examine factors associated with (1) screening completion and (2) positive screens.

**Results:**

Among 15 204 perioperative and procedural encounters (median age 14 [interquartile range 12, 16], 45% female), 13 566 (89.2%) had at least one ASQ item completed. Of these encounters, 494 (3.6%) had positive screens and 7 (0.1%) had screens indicating imminent risk. Adjusted odds of screening completion were lower among youth aged 10–12 than 13–15 years (adjusted odds ratio [aOR] 0.69, 95% CI 0.61–0.78) and those who preferred a language other than English or Spanish versus those who preferred English (aOR 0.55, 95% CI 0.41–0.75). Adjusted odds of positive screens were higher among females than males (aOR 2.49, 95% CI 2.06–3.03) and lower in ambulatory surgical centers than the children's hospital (aOR 0.26, 95% CI 0.14–0.42).

**Conclusions:**

Universal suicide risk screening can be implemented in perioperative and procedural areas, with positive screens in about 1 in 28 encounters. These settings represent an underutilized opportunity to identify at‐risk youth.

## 1 Introduction

Suicide is one of the leading causes of death among U.S. youth [1]. Among U.S. high school students in 2023, 20% reported seriously considering attempting suicide and 9% reported having attempted suicide during the past year [2]. Nevertheless, most youth who die by suicide do not have a prior diagnosis of a mental health condition, nor have they received care in traditional mental health settings [3].

Suicide risk screening conducted in non‐mental health service settings facilitates identification of youth with unmet mental health needs, enabling brief suicide prevention interventions, and linkage to services [4, 5]. Prior work has demonstrated successful integration of universal suicide risk screening in the emergency department and inpatient medical units, with some sites achieving screening rates that exceed 80% of eligible youth [6, 7].

Perioperative nurses likewise recognize the importance of detecting suicide risk among their patients. In a survey of nurses conducted after a brief educational session, all respondents agreed that youth aged 10 years and older should be screened for suicide risk in the perioperative setting [8]. While recognizing the importance of screening is an important first step, implementation of universal screening in the perioperative setting has not been previously reported.

In July 2022, our health system instituted a policy recommending universal suicide risk screening in perioperative and preprocedural areas using the Ask Suicide‐Screening Questions (ASQ), a brief 4–5 question screening instrument developed and validated to identify suicide risk in children ≥ 10 years old [9, 10]. Originally developed for use in emergency departments, the ASQ was designed to assess suicide risk for patients presenting for medical or surgical reasons. Since its development, the ASQ has been subsequently validated for use in outpatient clinics and inpatient medical units and has demonstrated high sensitivity and negative predictive value [7, 9, 11, 12]. In this study, we retrospectively evaluated the implementation of suicide risk screening in the perioperative and procedural areas of a pediatric health system by examining two outcomes: program reach (the proportion of eligible patients who were screened, i.e., screening completion rates) and screening positivity rates. We also assessed the sociodemographic and clinical characteristics associated with (1) screening completion and (2) positive screens and whether screening was maintained over time.

## 2 Methods

### 2.1 Study Design, Setting, and Selection of Encounters

We conducted a retrospective cross‐sectional study of universal suicide risk screening during perioperative and procedural encounters by youth 10–21 years old in an urban academic children's hospital and two affiliated ambulatory surgical centers using electronic health record data from August 2022 to February 2025. The study adhered to the Strengthening the Reporting of Observational Studies in Epidemiology (STROBE) guidelines (Appendix S1).

A health system policy, implemented in July 2022, states that all youth ≥ 10 years old should be screened for suicide risk using the Ask Suicide‐Screening Questions (ASQ), except for youth with intellectual disabilities that preclude understanding screening questions or an acute medical status that precludes an ability to respond to questions [9, 10]. Preteen suicide rates are rising in the US, and prior studies of the implementation of universal ASQ screening across health systems have demonstrated positivity rates of 2% for patients 10–11 years old, justifying the inclusion of preteens in screening workflows [13]. The ASQ was administered in the preoperative room as part of the routine preoperative nursing assessment. It was conducted privately to promote honest responses, with caregivers excluded unless the youth specifically requested their presence. Staff were encouraged to use standardized scripting provided by the National Institute of Mental Health [10]. In some instances, the ASQ may be completed via paper format, allowing youth to write their responses rather than answer verbally.

To our knowledge, no validated screening tools currently exist to assess suicide risk among youth with developmental disabilities, cognitive impairments, or communication disorders. Because the ASQ has not been validated in these populations, our institution employs an alternate, non‐validated screening process. In these situations, the parent or caregiver is asked: “Is your child demonstrating any self‐harming behaviors?” For youth who are unable to respond to questions due to their medical status, the clinician documents that suicide risk screening is unable to be assessed.

A positive screen occurs when the youth answers “Yes” to any of questions 1–5. Non‐imminent risk is defined as a “Yes” response to any of questions 1–4 only, while imminent risk is defined as a “Yes” response to question 5 (“Are you having thoughts of killing yourself right now?”). A positive screening result prompts immediate notification of a social worker or medical provider, who completes a Brief Suicide Safety Assessment to delineate the patient's level of risk [10]. If imminent risk is identified, patients are placed under continuous observation by appropriate hospital personnel and environmental safety precautions are implemented. Procedures are not canceled or delayed while awaiting further psychiatric evaluation. Following the procedure, if the youth otherwise meets criteria for discharge, the youth is transferred to the Emergency Department for further psychiatric evaluation. If the youth has a medical indication for admission following the procedure, psychiatric consultation is completed during the admission. Youth without imminent risk identified receive safety planning, including provision of mental health crisis hotlines and counseling to reduce access to lethal means, along with mental health referrals as indicated.

### 2.2 Measures

We defined an ASQ screen as “completed as recommended” when each of items 1–4 was marked Yes, No, or Refuse to Answer and, if any of items 1–4 were Yes, then item 5 was marked Yes, No, or Refuse to Answer [9, 10]. We defined an ASQ screen as “not completed” when each of items 1–5 was marked Unable to Assess or missing. We defined the remainder of ASQ screens as “partially complete.”

We defined a “complete alternative screening question” as Yes, No, or Refuse to Answer to the question, “Is your child demonstrating any self‐harming behaviors?” with each of the ASQ items 1–5 marked Unable to Assess or missing. We defined “workflow compliance” as either a complete ASQ or a complete alternative screening question.

We defined a “positive” ASQ screen as Yes or Refuse to Answer to any of items 1–5. We defined an “imminent positive” ASQ screen as Yes to item 5.

Additional study measures included age group (10–12, 13–15, 16–18, 19–21), sex (male, female), race and ethnicity (White, Black or African American, Hispanic/Latinx, Asian, and Other [including American Indian or Alaska Native, Native Hawaiian or Pacific Islander, and two or more races, collapsed because categories represented < 5% of the sample]), insurance (private, public, self‐pay/other), preferred language (English, Spanish, other), patient class (inpatient or observation status, outpatient), and screening location (operating room, procedural imaging, ambulatory surgical center). Age was defined as the age at the time of screening. Sex, race and ethnicity, insurance, and preferred language were extracted from patient demographics in the medical record. Screening location was determined by the department in which the ASQ was completed, and patient class was based on admission status at the time of the encounter.

### 2.3 Analysis

We described the percentage of encounters by youth 10–21 with any ASQ items completed (i.e., either partially complete, or completed as recommended), as well as the percentage of encounters with ASQ screens completed as recommended. Among encounters with any ASQ items completed, we described the percentage with positive screens and with screens that indicated imminent suicide risk. Among encounters with a positive ASQ, we described individual item responses. We examined monthly trends in completion of any ASQ items and in positivity rates across the study period.

The primary outcomes were (1) completion of suicide risk screening and (2) a positive suicide risk screen. Each outcome was modeled separately as the dependent variable in logistic regression analysis to assess demographic and clinical factors associated with these outcomes. We used both univariate and multivariable analysis to examine the association of demographic and clinical characteristics with (1) completion of any ASQ items, and (2) positive screens. In the multivariable analyses, covariates were age group, sex, race and ethnicity (collapsing any categories representing < 5% of the study sample), insurance, preferred language, patient class, and screening location. We followed the common rule of thumb for logistic regression requiring at least 10 outcome events per independent variable to avoid overfitting the data. Our dataset included 15 204 encounters with 494 events (i.e., positive ASQ screens), which was a sufficient sample size to include the seven selected covariates. The largest group in the study sample was set as the reference group. Records with missing data for any covariates were excluded from models. We also performed a sensitivity analysis to assess demographic and clinical characteristics associated with completion of the ASQ as recommended. Analyses were performed in R (version 4.3.3).

## 3 Results

### 3.1 Characteristics of Perioperative and Procedural Encounters

During the study period, 15 204 perioperative and procedural encounters occurred in youth aged 10–21 years. Encounters had a median age 14 (interquartile range 12, 16) and were 45% female, 52% privately insured, and 82% with English as the preferred language. The majority (82%) were outpatient surgery encounters. Most encounters were associated with operating room cases (73%), followed by procedural imaging (17%) and ambulatory surgical centers (6% and 4% occurred at each center, respectively) (Table 1).

**TABLE 1:** Characteristics of perioperative and procedural encounters by youth 10–21 years old, overall and by suicide risk screening completion.

Encounter characteristic	Overall, *N* (%)	ASQ completed	
		Any ASQ items completed^a^, *N* (%)	No ASQ items completed, *N* (%)
Overall *N*	15 204	13 566	1638
Age group			
10–12	4710 (31%)	4071 (30%)	639 (39%)
13–15	4988 (33%)	4498 (33%)	490 (30%)
16–18	4413 (29%)	4059 (30%)	354 (22%)
19–21	1093 (7.2%)	938 (6.9%)	155 (9.5%)
Sex			
Female	6876 (45%)	6228 (46%)	648 (40%)
Male	8328 (55%)	7338 (54%)	990 (60%)
Race and ethnicity			
Asian NH	703 (4.6%)	630 (4.6%)	73 (4.5%)
Black NH	1544 (10%)	1348 (9.9%)	196 (12%)
Hispanic/Latinx	5253 (35%)	4623 (34%)	630 (38%)
Two or more races	280 (1.8%)	244 (1.8%)	36 (2.2%)
White NH	6453 (42%)	5818 (43%)	635 (39%)
Other or not given^b^	971 (6.4%)	903 (6.7%)	68 (4.2%)
Insurance			
Private	7923 (52%)	7168 (53%)	755 (46%)
Public	7220 (47%)	6342 (47%)	878 (54%)
Self‐pay and other	61 (0.4%)	56 (0.4%)	5 (0.3%)
Preferred language			
English	12 481 (82%)	11 196 (83%)	1285 (78%)
Spanish	2372 (16%)	2078 (15%)	294 (18%)
Other languages	351 (2.3%)	292 (2.2%)	59 (3.6%)
Patient class			
Inpatient or observation status	2685 (18%)	2362 (17%)	323 (20%)
Outpatient surgery	12 519 (82%)	11 204 (83%)	1315 (80%)
Screening department			
Children's Hospital Operating Room	11 137 (73%)	9877 (73%)	1260 (77%)
Children's Hospital Procedural Imaging	2589 (17%)	2289 (17%)	300 (18%)
Ambulatory Surgical Center A	924 (6.1%)	914 (6.7%)	10 (0.6%)
Ambulatory Surgical Center B	554 (3.6%)	486 (3.6%)	68 (4.2%)

Abbreviations: ASQ, ask suicide‐screening questions; NH, non‐hispanic.

^a^ There were 13 000 encounters with a complete ASQ and 566 encounters with a partially complete ASQ.

^b^ Individuals identifying as American Indian or Pacific Islander were grouped with “Other or not given” due to small sample size.

### 3.2 Ask Suicide‐Screening Questions: Completion and Positivity Rates

Among all perioperative and procedural encounters, 13 566 (89.2%) had any ASQ items completed, of which 13 000 (95.8%) had all items completed as recommended. Of the 13 566 encounters with any ASQ items completed, 494 (3.6%) had a positive screen and 7 (0.1%) had a screen indicating imminent risk (Tables 2 and 3). Of the 494 encounters with a positive screen, 300 (61%) reflected a lifetime history of a suicide attempt only (i.e., Yes to ASQ item 4 only) with no recent suicidal ideation (Table 3).

**TABLE 2:** Characteristics of perioperative encounters with suicide risk screening performed, overall and by screening results.

Encounter characteristic	Overall^a^, *N* (%)	ASQ positive	
		Yes, *N* (%)	No, *N* (%)
Overall *N*	13 566	494	13 072
Age group			
10–12	4071 (30%)	91 (18%)	3980 (30%)
13–15	4498 (33%)	168 (34%)	4330 (33%)
16–18	4059 (30%)	204 (41%)	3855 (29%)
19–21	938 (6.9%)	31 (6.3%)	907 (6.9%)
Sex			
Female	6228 (46%)	333 (67%)	5895 (45%)
Male	7338 (54%)	161 (33%)	7177 (55%)
Race and ethnicity			
Asian NH	630 (4.6%)	20 (4.0%)	610 (4.7%)
Black NH	1348 (9.9%)	48 (9.7%)	1300 (9.9%)
Hispanic/Latinx	4623 (34%)	168 (34%)	4455 (34%)
Two or more races	244 (1.8%)	13 (2.6%)	231 (1.8%)
White NH	5818 (43%)	208 (42%)	5610 (43%)
Other or Not Given^b^	903 (6.7%)	37 (7.5%)	866 (6.6%)
Insurance			
Private	7168 (53%)	247 (50%)	6925 (53%)
Public	6342 (47%)	243 (49%)	6095 (47%)
Self‐pay and other	56 (0.4%)	4 (0.8%)	52 (0.4%)
Language			
English	11 196 (83%)	421 (85%)	10 775 (82%)
Spanish	2078 (15%)	65 (13%)	2013 (15%)
Other languages	292 (2.2%)	8 (1.6%)	284 (2.2%)
Patient class			
Inpatient or observation status	2362 (17%)	113 (23%)	2249 (17%)
Outpatient surgery	11 204 (83%)	381 (77%)	10 823 (83%)
Screening department			
Children's Hospital Operating Room	9877 (73%)	443 (90%)	9434 (72%)
Children's Hospital Procedural Imaging	2289 (17%)	37 (7.5%)	2252 (17%)
Ambulatory Surgical Center A	914 (6.7%)	11 (2.2%)	903 (6.9%)
Ambulatory Surgical Center B	486 (3.6%)	3 (0.6%)	483 (3.7%)

Abbreviation: NH, non‐hispanic.

^a^ There were 13 000 encounters with a complete ASQ and 566 encounters with a partially complete ASQ.

^b^ Individuals identifying as American Indian or Pacific Islander were grouped with “Other or not given” due to small sample size.

**TABLE 3:** Individual item responses among perioperative encounters with a positive suicide risk screen.

Ask suicide‐screening question	*N* (%)
Q1: “In the past few weeks, have you wished you were dead?”	
Yes	103 (22%)
No	367 (74%)
Unable to assess	0 (0%)
Refuse to answer	17 (3.4%)
Missing	2 (0.4%)
Q2: “In the past few weeks, have you felt that you or your family would be better off if you were dead?”	
Yes	100 (20%)
No	372 (75%)
Unable to assess	1 (0.2%)
Refuse to answer	18 (3.6%)
Missing	3 (0.6%)
Q3: “In the past week, have you been having thoughts about killing yourself?”	
Yes	64 (13%)
No	405 (82%)
Unable to assess	2 (0.4%)
Refuse to answer	20 (4.0%)
Missing	3 (0.6%)
Q4: “Have you ever tried to kill yourself?”	
Yes	345 (70%)
No	124 (25%)
Unable to assess	2 (0.4%)
Refuse to answer	20 (4.0%)
Missing	3 (0.6%)
Q5: “Are you having thoughts of killing yourself right now?”^a^	
Yes	7 (1.4%)
No	458 (93%)
Unable to assess	1 (0.2%)
Refuse to answer	12 (2.4%)
Missing	16 (3.2%)
Yes to Q4 only^b^	300 (61%)

^a^ Q5 is only intended to be asked if there is a Yes response to any of Q1‐4.

^b^ Defined as Yes to Q4 along with a response of No, Refuse to Answer, Unable to Assess, or missing for Q1, Q2, Q3, and Q5.

### 3.3 Alternative Screening Question for Children With Intellectual Disabilities

Among all perioperative and procedural encounters, 1542 (10.1%) had a completed alternative question for children with intellectual disabilities who were unable to understand ASQ questions. Of these, 7 (0.5%) had a positive response. Among all perioperative and procedural encounters, 14 542 (95.6%) encounters demonstrated workflow compliance, as indicated by either a complete ASQ or a complete alternative screening question.

### 3.4 Characteristics Associated With Completion of Ask Suicide‐Screening Questions

Six encounters were excluded from models due to missing sex. In logistic regression models adjusted for demographic and clinical characteristics, there were lower adjusted odds of ASQ completion among encounters by 10–12 year‐olds (adjusted odds ratio [aOR] 0.69, 95% CI 0.61–0.78) and 19–21‐year‐olds (aOR 0.66, 95% CI 0.54–0.80) compared to 13–15‐year‐olds; among encounters with public insurance (aOR 0.83, 95% CI 0.73–0.95) compared to private insurance, and among encounters with a preferred language other than English or Spanish (aOR 0.55, 95% CI 0.41–0.75) compared to a preferred language of English. In logistic regression models adjusted for demographic and clinical characteristics, there were higher adjusted odds of ASQ completion among encounters by girls (aOR 1.29, 95% CI 2.06–3.03) than boys, and in ambulatory surgical centers (aOR 2.18, 95% CI 0.14–0.42) relative to the main children's hospital (Table 4).

**TABLE 4:** Univariate and multivariable regression: characteristics associated with ASQ completion in the perioperative setting.

Encounter characteristic	Odds of ASQ completion,^a^ OR (95% CI)	Adjusted odds of ASQ completion,^a^ aOR (95% CI)
Age group		
10–12	0.69 (0.61, 0.79)	0.69 (0.61, 0.78)
13–15	Reference	Reference
16–18	1.25 (1.08, 1.44)	1.26 (1.09,1.46)
19–21	0.66 (0.54, 0.80)	0.66 (0.54,0.80)
Sex		
Female	1.30 (1.17, 1.44)	1.29 (1.16, 1.44)
Male	Reference	Reference
Race and ethnicity		
Black NH	0.75 (0.63, 0.89)	0.86 (0.72, 1.04)
Hispanic/Latinx	0.80 (0.71, 0.90)	0.91 (0.78, 1.06)
White NH	Reference	Reference
Other or not given^b^	1.10 (0.92, 1.31)	1.27 (1.06, 1.53)
Insurance		
Private	Reference	Reference
Public	0.76 (0.69, 0.84)	0.83 (0.73, 0.95)
Self‐pay/other	1.18 (0.51, 3.39)	1.33 (0.58, 3.87)
Language		
English	Reference	Reference
Spanish	0.81 (0.71, 0.93)	0.96 (0.81, 1.14)
Other languages	0.57 (0.43, 0.76)	0.55 (0.41, 0.75)
Patient class		
Inpatient/observation status	0.86 (0.75, 0.98)	0.87 (0.77, 1.00)
Outpatient surgery	Reference	Reference
Screening department		
Children's Hospital	Reference	Reference
Ambulatory Surgical Center	2.30 (1.83, 2.93)	2.18 (1.74, 2.79)

Abbreviation: NH, non‐hispanic.

^a^ Defined as complete or partially complete ASQ screen.

^b^ Other includes non‐Hispanic American Indian/Alaska Native, Asian, Pacific Islander, and Two or more races, which were analyzed together due to small sample size.

### 3.5 Characteristics Associated With Positive Response to Ask Suicide‐Screening Questions

There were lower adjusted odds of a positive screen among 10–12‐year‐olds (aOR 0.59, 95% CI 0.45–0.77) and higher adjusted odds of a positive screen among 16–18‐year‐olds (aOR 1.33, 95% CI 1.08–1.64) compared to 13–15‐year‐olds. There were higher adjusted odds of positive screens among females (aOR 2.49, 95% CI 2.06–3.03) compared to males and among encounters with public insurance (aOR 1.30, 95% CI 1.04–1.62) compared to private insurance. There were lower adjusted odds of positive screens in the ambulatory surgical centers compared to the children's hospital (aOR 0.26, 95% CI 0.14–to 0.42) (Table 5).

**TABLE 5:** Univariate and multivariable regression: characteristics associated with a positive suicide risk screen among perioperative encounters with any screening items completed.

Encounter characteristic	Odds of positive screen, OR (95% CI)	Adjusted odds of positive screen, aOR (95% CI)
Age group		
10–12	0.59 (0.45, 0.76)	0.59 (0.45, 0.77)
13–15	Reference	Reference
16–18	1.36 (1.11, 1.68)	1.33 (1.08, 1.64)
19–21	0.88 (0.59, 1.28)	0.80 (0.53, 1.17)
Sex		
Female	2.52 (2.08, 3.06)	2.49 (2.06, 3.03)
Male	Reference	Reference
Race and ethnicity		
Black NH	1.00 (0.72, 1.36)	0.92 (0.65, 1.28)
Hispanic/Latinx	1.02 (0.83, 1.25)	1.00 (0.77, 1.30)
White NH	Reference	Reference
Other or not given^a^	1.11 (0.83, 1.45)	1.10 (0.82, 1.45)
Insurance		
Private	Reference	Reference
Public	1.15 (0.96, 1.38)	1.30 (1.04, 1.62)
Self‐pay/other	2.19 (0.66, 5.41)	2.14 (0.63, 5.44)
Language		
English	Reference	Reference
Spanish	0.83 (0.63, 1.07)	0.69 (0.50, 0.95)
Other languages	0.72 (0.33, 1.37)	0.59 (0.26, 1.16)
Patient class		
Inpatient/observation status	1.43 (1.15, 1.76)	1.20 (0.96, 1.49)
Outpatient surgery	Reference	Reference
Screening department		
Children's Hospital	Reference	Reference
Outpatient Surgical Center	0.25 (0.14, 0.40)	0.26 (0.14, 0.42)

Abbreviation: NH, non‐hispanic.

^a^ Other includes non‐Hispanic American Indian/Alaska Native, Asian, Pacific Islander, and Two or more races, which were analyzed together due to small sample size.

### 3.6 Sensitivity Analysis Examining Completion of All Questions as Recommended

When examining the subgroup of encounters with all ASQ questions completed as recommended, model results were similar except that the adjusted odds of a positive screen were no longer significant for encounters with public insurance relative to private insurance, while the adjusted odds of a positive screen were significantly higher for inpatient surgical encounters relative to ambulatory surgical encounters (Supplemental Table S1).

### 3.7 Monthly Rates of Completion of Any ASQ Items and Monthly ASQ Positivity Rates

**FIGURE 1:**
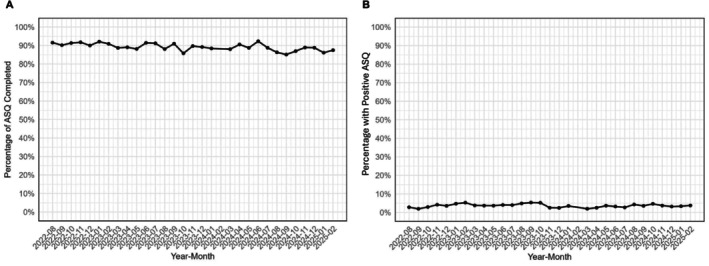
ASQ completion and positivity rates over time. Panel A. Monthly rates of completion of any Ask Suicide‐Screening Question (ASQ) items during perioperative and procedural encounters by youth 10–12 from August 2022 to February 2025. Panel B. Monthly ASQ positivity rates, among perioperative and procedural encounters with any ASQ items completed, from August 2022 to February 2025.

Over the study period, monthly ASQ completion rates during perioperative or procedural encounters remained consistently high (≥ 85%), and monthly ASQ positivity rates were similarly stable over time (Figure 1).

## 4 Discussion

This study demonstrates that universal suicide risk screening can be successfully integrated and sustained in the perioperative and procedural settings within a pediatric health system, and that screening completion and positive screen results vary by demographic and clinical characteristics. The program achieved broad reach with robust completion rates and consistent adherence to the full screening protocol over two years. Sustained performance is notable, as prior studies have shown that new health care interventions, such as screening programs, often experience declining uptake over time once initial implementation efforts wane [14, 15]. These findings suggest that perioperative settings can effectively support standardized suicide risk screening protocols.

The overall screen positivity rate of 3.6%, occurring in approximately 1 in 28 encounters, aligns with rates reported in emergency and inpatient settings [6, 16]. Notably, most positive screens reflected a past suicide attempt without current ideation, indicating underlying mental health needs rather than an acute crisis. The low rate of imminent risk identified in this setting may reassure institutions considering similar efforts, particularly in ambulatory surgical centers, where on‐site mental health resources may be limited.

Screening completion varied by age, language, and site of care, revealing disparities that warrant attention. Screening was less likely to be completed during encounters by younger adolescents. While the reasons are unclear, this may reflect staff misperceptions that preteens are at lower risk for suicide [17] and therefore prioritize screening less, even unconsciously. Preprocedural visits are time‐constrained and staff may omit the screening for younger patients if they perceive the added burden unnecessary. Staff may also feel less comfortable asking suicide‐related questions to younger children. Completion was also lower among patients whose preferred language was neither English nor Spanish, which may suggest systemic barriers, such as limited interpreter infrastructure. Completion rates were higher in ambulatory surgical centers compared to the main children's hospital. Contributing factors may include increased staffing ratios and lower patient volume at ambulatory surgical centers, enabling more time for screening. Additionally, these youth may have decreased medical complexity, a lower prevalence of intellectual disabilities, and a less intensive preprocedural preparation process, which may facilitate screening. Screening positivity rates varied by demographic and clinical characteristics, providing insights into which youth may be at greater risk. Girls were more likely to screen positive than boys, consistent with prior literature indicating higher rates of suicidal thoughts and attempts among girls [18]. We also found that youth with public insurance were more likely to screen positive compared to those with private insurance, reflecting broader socioeconomic disparities in mental health care [19, 20]. Additionally, youth seen at the main children's hospital were more likely to screen positive than those in ambulatory centers, likely reflecting differences in medical complexity and psychosocial burden. Recognizing these patterns can help clinicians prioritize mental health screening and follow‐up for populations at elevated risk.

This study has several limitations. First, we recognize that there may be additional unmeasured confounders, such as psychosocial or clinical characteristics not captured, that could influence screening completion or positivity. Further, we assessed screening positivity but not whether youth were already receiving mental health care, nor were we able to ascertain how often positive screens led to interventions or referrals. Also, provider‐level variability in how the ASQ was administered could have influenced results, and we did not evaluate perceptions of screening acceptability among youth, caregivers, or staff. Prior work shows that ASQ screening is highly acceptable to patients, parents, and staff [7, 21]. However, that work was conducted in outpatient clinics and hospital settings rather than the perioperative environment. Because our study relied on electronic health record data, we were unable to capture participants' perspectives or comfort levels in answering suicide‐related questions, nor could we assess the impact of screening on perioperative workflows, case delays, or long‐term outcomes such as actual suicide attempts.

Despite these limitations, this study highlights an opportunity for perioperative teams, including anesthesiologists, to contribute meaningfully to youth suicide prevention. Integrating suicide risk screening into routine care can identify youth with unmet mental health needs and connect them with needed care. Specifically, screening in the perioperative setting is particularly well suited to identify mental health concerns that may be exacerbated by preoperative anxiety about the surgical procedure, postoperative pain, impaired mobility, changes to routines, or decreased independence [22]. Screening also enables identification of youth at risk for suicide by medication overdose, which is an important consideration when prescribing new medications, including opioids, postoperatively. Risk identification can prompt counseling on secure medication storage, a recommended suicide prevention practice [23].

For institutions or anesthesiologists seeking to implement universal suicide risk screening in perioperative settings, several practical considerations are important to consider. Staff education is essential to promote sensitive and consistent administration of the screening questions in a supportive, nonjudgmental manner. Incorporating the questionnaire into existing workflows can minimize disruption to patient flow. Clear protocols should be established for managing positive screens, including access to mental health professionals. Securing institutional and leadership support can facilitate adequate resource allocation, interdisciplinary collaboration, and sustained adoption.

To strengthen follow‐up and continuity of care, perioperative teams may benefit from closer collaboration with behavioral health personnel, such as through the establishment of a dedicated perioperative social work role to provide real‐time support, facilitate timely referrals, and coordinate care. In addition, developing electronic health record–based clinical decision support tools that automatically flag positive screens and initiate standardized referral pathways can enhance care coordination and reduce gaps in follow‐up care. Our findings suggest additional opportunities to concentrate resources and improve equity in screening. For instance, concentrating behavioral health and social work support at the main hospital, where screening positivity was highest, may improve referral processes and follow‐up outcomes. Similarly, expanding language interpretation infrastructure may help ensure equitable screening and effective communication for patients who prefer a language other than English.

While implementation in our health system was successful, additional studies are needed to further optimize and understand the benefits of suicide risk screening in the perioperative setting. Future work should explore patient, family, and staff perspectives; refine practices to improve fidelity in screening; track referral follow‐through and patient outcomes over time.

## Author Contributions

Michelle Tsao and Jennifer A. Hoffmann provided substantial contributions to study conception and design, interpretation of data, and drafting of the manuscript. Chunyi Wu provided substantial contributions to analysis of data and revised the article critically for important intellectual content. Arkadeep Ghosh, Amanda N. Burnside, Matthew J. Rowland, and Eric Cheon provided substantial contributions to study design, interpretation of data, and revised the article critically for important intellectual content. All authors approved the final manuscript as submitted and agreed to be accountable for all aspects of the work.

## Funding

This work was supported by the National Institutes of Health (K23MH135206‐01).

## Ethics Statement

This study was conducted in accordance with ethical standards, and the Institutional Review Board (IRB) at the lead author's hospital reviewed and approved this study. Protected health information confidentiality was strictly maintained, and data were anonymized to protect personal information.

## Consent

The authors have nothing to report.

## Conflicts of Interest

The authors declare no conflicts of interest.

## Supporting information


**Appendix S1:** STROBE reporting guideline.

**Table S1:** Univariate and multivariable regression: characteristics associated with a positive suicide among perioperative encounters with screening completed as recommended—sensitivity analysis.

## Data Availability

The data that support the findings of this study are available from the corresponding author upon reasonable request.

## References

[1] Centers for Disease Control and Prevention , “Leading Causes of Death and Injury,” accessed May 21, 2023, https://wisqars.cdc.gov/.

[2] Centers for Disease Control and Prevention , Youth Risk Behavior Survey Data Summary & Trends Report: 2013–2023 (U.S. Department of Health and Human Services, 2024).

[3] K. Rice , M. Brown , N. Nataraj , and L. Xu , “Circumstances Contributing to Suicide Among U.S. Adolescents Aged 10–19 Years With and Without a Known Mental Health Condition: National Violent Death Reporting System, 2013–2018,” Journal of Adolescent Health 72 (2023): 1–7. 10.1016/j.jadohealth.2022.11.009 PMC10033346 36623968

[4] K. Alrisi , N. Alnasif , A. Nazeer , J. Shareef , and F. Latif , “Risk of Suicide in Children and Adolescents in the Emergency Department‐Is Universal Screening the Answer?,” Archives of Disease in Childhood 108, no. 12 (2023): 970–974. 36927622 10.1136/archdischild-2022-325122

[5] S. K. Doupnik , B. Rudd , T. Schmutte , et al., “Association of Suicide Prevention Interventions With Subsequent Suicide Attempts, Linkage to Follow‐Up Care, and Depression Symptoms for Acute Care Settings: A Systematic Review and Meta‐Analysis,” JAMA Psychiatry 77, no. 10 (2020): 1021–1030. 32584936 10.1001/jamapsychiatry.2020.1586 PMC7301305

[6] P. E. Cervantes , D. E. M. M. Seag , A. Baroni , et al., “Universal Suicide Risk Screening for Youths in the Emergency Department: A Systematic Review,” Psychiatric Services 73, no. 1 (2022): 53–63. 34106741 10.1176/appi.ps.202000881 PMC8655012

[7] L. M. Horowitz , E. A. Wharff , A. M. Mournet , et al., “Validation and Feasibility of the ASQ Among Pediatric Medical and Surgical Inpatients,” Hospital Pediatrics 10, no. 9 (2020): 750–757. 32826283 10.1542/hpeds.2020-0087 PMC7446546

[8] K. Mancinelli‐Hough , K. Lucas Breda , C. Karl , and B. A. Wentland , “Don't Ask, Won't Tell: Suicide Screening in the Pediatric Perioperative Setting,” Comprehensive Child and Adolescent Nursing 45, no. 4 (2022): 395–402. 36440865 10.1080/24694193.2022.2060376

[9] L. M. Horowitz , J. A. Bridge , S. J. Teach , et al., “Ask Suicide‐Screening Questions (ASQ): A Brief Instrument for the Pediatric Emergency Department,” Archives of Pediatrics & Adolescent Medicine 166, no. 12 (2012): 1170–1176. 23027429 10.1001/archpediatrics.2012.1276 PMC6889955

[10] National Institute of Mental Health , “Ask Suicide‐Screening Questions Toolkit,” accessed July 30, 2025, https://www.nimh.nih.gov/research/research‐conducted‐at‐nimh/asq‐toolkit‐materials/index.shtml.

[11] L. D. Aguinaldo , S. Sullivant , E. C. Lanzillo , et al., “Validation of the Ask Suicide‐Screening Questions (ASQ) With Youth in Outpatient Specialty and Primary Care Clinics,” General Hospital Psychiatry 68 (2021): 52–58, 10.1016/j.genhosppsych.2020.11.006. 33310014 PMC7855604

[12] K. Roaten , L. M. Horowitz , J. A. Bridge , et al., “Universal Pediatric Suicide Risk Screening in a Health Care System: 90,000 Patient Encounters,” Journal of the Academy of Consultation‐Liaison Psychiatry 62, no. 4 (2021): 421–429. 34219656 10.1016/j.jaclp.2020.12.002

[13] D. A. Ruch , L. M. Horowitz , J. L. Hughes , et al., “Suicide in US Preteens Aged 8 to 12 Years, 2001 to 2022,” JAMA Network Open 7, no. 7 (2024): e2424664, 10.1001/jamanetworksopen.2024.24664. 39078634 PMC11289692

[14] J. Braithwaite , K. Ludlow , L. Testa , et al., “Built to Last? The Sustainability of Healthcare System Improvements, Programmes and Interventions: A Systematic Integrative Review,” BMJ Open 10, no. 6 (2020): e036453. 10.1136/bmjopen-2019-036453 PMC7265014 32487579

[15] J. M. Boggs , J. Richards , G. Simon , et al., “Suicide Screening, Risk Assessment, and Lethal Means Counseling During Zero Suicide Implementation,” Psychiatric Services 75, no. 7 (2024): 638–645, 10.1176/appi.ps.20230211. 38566561 PMC11404670

[16] A. Lund , K. Denicolo , S. Tomko , et al., “Improving Universal Suicide Risk Screening Rates at a Children's Hospital,” Pediatrics 155, no. 5 (2025): e2024065901. 40180330 10.1542/peds.2024-065901 PMC12640608

[17] E. C. Lanzillo , L. M. Horowitz , E. A. Wharff , A. H. Sheftall , M. Pao , and J. A. Bridge , “The Importance of Screening Preteens for Suicide Risk in the Emergency Department,” Hospital Pediatrics 9, no. 4 (2019): 305–307. 30858170 10.1542/hpeds.2018-0154 PMC6434973

[18] L. L. Hua , J. Lee , M. H. Rahmandar , E. J. Sigel , Committee on Adolescents , and Council on Injury, Violence, and Poison Prevention , “Suicide and Suicide Risk in Adolescents,” Pediatrics 153, no. 1 (2024): e2023064800. 38073403 10.1542/peds.2023-064800

[19] J. A. Hoffmann , C. A. Farrell , M. C. Monuteaux , E. W. Fleegler , and L. K. Lee , “Association of Pediatric Suicide With County‐Level Poverty in the United States, 2007–2016,” JAMA Pediatrics 174, no. 3 (2020): 287–294. 31985759 10.1001/jamapediatrics.2019.5678 PMC6990805

[20] R. Mojtabai and M. Olfson , “National Trends in Mental Health Care for US Adolescents,” JAMA Psychiatry 77, no. 7 (2020): 703–714. 32211824 10.1001/jamapsychiatry.2020.0279 PMC7097842

[21] M. V. Tipton , M. N. F. Arruda‐Colli , S. Z. Bedoya , M. Pao , and L. Wiener , “The Acceptability of Screening for Suicide Risk Among Youth in Outpatient Medical Settings: Child and Parent Perspectives,” Journal of Psychosocial Oncology 39, no. 6 (2021): 789–795, 10.1080/07347332.2020.1856997. 33306002 PMC8192582

[22] R. H. Dustin and B. A. Winters , “Recommendations for Perioperative Care of Adolescents at Risk for Suicide,” Journal of Perioperative Practice 32, no. 4 (2022): 69–73. 34380352 10.1177/17504589211020905

[23] C. W. Runyan , A. Becker , S. Brandspigel , C. Barber , A. Trudeau , and D. Novins , “Lethal Means Counseling for Parents of Youth Seeking Emergency Care for Suicidality,” Western Journal of Emergency Medicine 17 (2016): 8–14. 26823923 10.5811/westjem.2015.11.28590 PMC4729425

