# Supplementary material for: Retrospective Evaluation of the Implementation of Universal Suicide Risk Screening for Youth in the Perioperative and Procedural Areas of a Health System
Source: Paediatr Anaesth. 2026 Jan 14;36(4):421–30. doi: 10.1002/pan.70127 (PMC12805949; doi:10.1002/pan.70127)
Supplement: Supplementary file 2 — Table S1: Univariate and multivariable regression: characteristics associated with a positive suicide among perioperative encounters with screening completed as recommended—sensitivity analysis. [file PAN-36-421-s001.docx]

**Supplemental Table 1. Univariate and Multivariable Regression: Characteristics Associated with a Positive Suicide among Perioperative Encounters with Screening Completed as Recommended – Sensitivity Analysis**

| **Encounter Characteristic** | Odds of Positive Screen,   OR (95% CI) | Adjusted Odds   of Positive Screen,   aOR (95% CI) |
| --- | --- | --- |
| Age group    10-12    13-15    16-18    19-21 | 0.49 (0.37,0.65)  Reference  1.37 (1.10,1.69)  0.90 (0.59,1.32) | 0.49 (0.37,0.65)  Reference  1.33 (1.07,1.65)  0.81 (0.54,1.19) |
| Sex    Female    Male | 2.61 (2.14,3.20)  Reference | 2.59 (2.13,3.18)  Reference |
| Race and Ethnicity    Black NH    Hispanic/Latinx    White NH    Other or Not Given^†^ | 1.05 (0.75,1.44)  1.06 (0.85,1.31)  Reference  1.11 (0.83,1.47) | 0.95 (0.66,1.34)  1.05 (0.80,1.37)  Reference  1.11 (0.82,1.49) |
| Insurance    Private    Public    Self-Pay/Other | Reference  1.21 (1.00,1.45)  2.47 (0.74,6.12) | Reference  1.37 (1.09,1.72)  2.47 (0.73,6.32) |
| Language    English    Spanish    Other Languages | Reference  0.80 (0.60,1.04)  0.58 (0.23,1.19) | Reference  0.63 (0.45,0.87)  0.47 (0.18,1.01) |
| Patient Class    Inpatient/Observation Status    Outpatient Surgery | 1.40 (1.12,1.75)  Reference | 1.19 (0.94,1.48)  Reference |
| Screening Department    Children’s Hospital    Outpatient Surgical Center | Reference  0.28 (0.16,0.46) | Reference  0.30 (0.17,0.49) |

  NH: Non-Hispanic

^†^ Other includes non-Hispanic American Indian/Alaska Native, Asian, Pacific Islander, and Two or more races, which were analyzed together due to small sample size.
